# The Impact of United Kingdom and Malaysia's Inherent Health Systems on Their COVID‐19 Responses: A Comparison of Containment Strategies

**DOI:** 10.1002/wmh3.412

**Published:** 2021-05-04

**Authors:** Shereen Allaham, Isabel‐Cathérine Demel, Intesar Nur, Faizul Nizam Abu Salim, Logan Manikam

**Keywords:** COVID‐19, community spread, inherent health system, pandemic response, public health

## Abstract

In March 2020, the outbreak of COVID‐19 was officially declared a global pandemic by the World Health Organization. Given the novelty of the virus, and hence, lack of official guidance on effective containment strategies, individual countries opted for different containment approaches ranging from herd immunity to strict lockdown. The opposing strategies followed by the United Kingdom and its former colony, Malaysia, stand exemplary for this. Real‐time polymerase chain reaction was implemented for testing in both counties. Malaysia acted with strict quarantining rules and infection surveillance. The United Kingdom followed an initially lenient, herd‐immunity approach with strict lockdown only enforced weeks later. Although based on the same health‐care structure historically, Malaysia developed a more unified health system compared with the United Kingdom. We suggest that this more centralized structure could be one possible explanation for why Malaysia was able to react in a more timely and efficient manner, despite its closer geographic proximity to China. We further explore how the differences in testing and quarantining strategy, as well as political situation and societal compliance could account for the discrepancy in the United Kingdom's versus Malaysia's relative success of COVID‐19 containment.

## Key Points

1. Different countries employed a range of strategies toward the COVID‐19 pandemic, with varying levels of success.
2. Malaysia's strict rule enforcement were reflected both in quantitative and qualitative terms.
3. Despite the closer geographical proximity to the source of the outbreak, Malaysia fared better than the United Kingdom in its response to the pandemic, suggesting that there are valuable lessons to be learned for the developed from developing, limited‐resource countries.

## Introduction

The first case of COVID‐19 was officially reported by the Chinese authorities on December 31, 2019. Three months later, on March 11, 2020, the outbreak was declared a global pandemic by the World Health Organization, 2020c). Given the novelty of the SARS‐COV2 virus and the unprecedented nature of the situation, little to no official guidelines were available to advise governments on the most effective containment strategy. Countries were thus forced to react on a national and local, rather than global level, some opting for herd immunity with voluntary social distancing, while others enforced prompt lockdown with intense infection surveillance.

The varying approaches employed by the United Kingdom and its former colony, Malaysia, stand exemplary for this: despite similarities in their historic health‐care structure and recent political situation at the time of the outbreak, the two countries fared differently during the pandemic. The ultimate success, and long‐term consequences of their approach, that is, from a social and economic perspective, remains to be determined (Gibney, 2020).

The aim of this paper is to compare and contrast their containment approaches by shedding light on factors that facilitated—or hindered—the combatting of the COVID‐19 pandemic. Using the case study of the United Kingdom versus Malaysia, we explore the role their health systems and organizational structure, pandemic preparedness, and response, as well as governance and societal compliance, have played in shaping the overall impact of the pandemic on their respective population.

### Health Systems—The United Kingdom Versus Malaysia

The United Kingdom funded the National Health Service (NHS) in 1948 with the primary objective of offering treatment to everyone irrespective of their ability to pay (Triggle, 2018). The NHS is subdivided into four different countries (England, Scotland, Wales, and Northern Ireland) each of which controls the specific individual and public health‐care policies for that respective area (Boyle, 2011). In the United Kingdom, different independent organizations are responsible for the delivery of numerous facets of health care. For example, Public Health England (PHE) oversees the public health component, while the NHS manages clinical and health‐related services, and the Department of Health and Social Care (DHSC) coordinates government policy on health and adult social care matters in England (National Healthcare Service, 2013).

As a former British colony, Malaysia “inherited” the British health‐care system. Malaysia, however, has since followed—or maintained—a more unified health‐care system approach. The country's main provider of health‐care services, the Ministry of Health (MOH), can effectuate changes from a National to a District level (Thomas, Beh, & Nordin, 2011 Ministry of Health Malaysia, 2019). The local system hence remained very centralized at the Federal Government level, with the MOH asserting control over State Health Department at 13 states and 3 federal territories. At the federal level, the Director‐General of Health (DG) is assisted by three deputies namely the Medical Deputy DG (similar to NHS National Medical Director), Public Health Deputy DG (similar to PHE), and Research and Technical Support Deputy DG (who oversee six MOH research institutes and the planning division, mimicking DHSC to a certain extent). Furthermore, Malaysia has both a public and a private health sector. Rather than paying health‐care‐related services for everyone, the Malaysian approach prioritizes spending on essential care and promotes equity by subsidizing treatments those who cannot afford it. This is associated with a reduction in the government's burden, leading to Malaysia only allocating around 4.24 percent of GDP on health care—less than half compared with the United Kingdom (Cooper, 2020; Ministry of Health Malaysia, 2019).

### Pandemic Preparedness, COVID‐19 Arrival and Response: The United Kingdom Versus Malaysia

The first case of COVID‐19 was discovered in the United Kingdom on January 29 (Gallagher, 2020) compared with January 24, 2020, in Malaysia (World Health Organization, 2020a), where the first surge of COVID‐19 infections likely originated from a group of Chinese travelers entering the country via Singapore. As an early preventive measure, a stringent screening process was imposed at all Malaysian airports given the geographic proximity to the source of the outbreak (Shah et al., 2020). With this strict approach, the initial wave was contained successfully, counting only 22 cases over a span of 3 weeks ending February 16, 2020 (World Health Organization, 2020b). A second case surge was noted on February 27, overlapping with a religious gathering in Seri Petaling Mosque attended by 16,000 individuals (DG of Health,2020a). This likely had significant consequences on Malaysia's infection numbers and its overall performance in containing the pandemic. Malaysia's first fatal case, reported on March 17, 2020, was also found to be related to this religious gathering (Shah et al., 2020).

Subsequently, the Malaysian government, in collaboration with the MOH, focused considerable efforts into containing the outbreak. Participants who had attended the gathering at Seri Petaling Mosque were urged to undergo screening. The resources available to private and public hospitals were enhanced and local bed availability for COVID‐19 cases was increased. On March 1, an alliance involving 38 professional medical societies was established with the aim of keeping the population well‐informed with accurate and up‐to‐date guidance (Shah et al., 2020).

Furthermore, Malaysia instated a Movement Control Order (MCO) on March 18, 2020 after the official classification of COVID‐19 as a global pandemic (*New Straits Times*, 2020). This encompassed regulations on international and interstate traveling, shop operating, schools and universities opening, as well as the cancellation of worship services (Shah et al., 2020; World Health Organization, 2020c). With regards to funding, the MHO set up an action coalition to obtain financial aid from companies, NGOs, and other organizations to fight the outbreak (Shah et al., 2020).

Malaysia's rapid response and strict enforcement of rules had imperative value, especially when accounting for COVID‐19's mode of transmission (respiratory droplet and direct contact, and consequently, the importance of reducing human‐to‐human contact in its containment) (Public Health England, 2020).

By contrast, the United Kingdom followed a different, more lenient approach. Although the PHE mentioned their 5‐year plan to tackle future pandemics in 2019, specifically referring to the United Kingdom's ability of “Test and Trace” for MERS‐COV and its successes, the procedure for an outbreak of an unknown disease was not mentioned (Public Health England, 2019). Similarly, the issue around access to and availability of personal protective equipment remains untouched upon.

Consequently, the United Kingdom found itself poorly prepared with the third‐lowest number of hospital beds per 1,000 population among the Group of 20 countries (Organisation for Economic Co‐operation and Development, 2020). Irrespective of that, the country initially opted for a “herd immunity” approach due to a concern about the economic and social consequences, for example, loneliness, isolation, lack of health‐care access, of a strict lockdown situation (Hunter, 2020). A change in government advice followed on March 16, as cases requiring ICU admission started exceeding the NHS’ capacities: Symptomatic individuals and their close household contacts were advised to self‐isolate for 7 days, with anyone over 70 to avoid any “non‐essential social contact” (Hunter, 2020). Mass gatherings were discouraged, but not banned. The hard lockdown was enforced on March 23, 2020. By this time, 6,650 people had been confirmed positive for the disease with 335 fatalities (Rawlinson, 2020) compared to only 2 deaths and 673 cases in Malaysia on March 17 (DG of Health, 2020b). Thorough preparation, a timely and stricter response, as well as consistent government advice would likely have lessened United Kingdom infections and deaths.

### Approach to “Test and Trace”: The United Kingdom Versus Malaysia

What both countries share is that real‐time reverse‐transcription polymerase chain reaction (RT‐PCR) (Watson, Whiting, & Brush, 2020) was employed in the detection and diagnosis of COVID‐19 infected individuals. Although this is the WHO‐recommended gold standard, RT‐PCR requires big laboratories, hence being subject to limitations in scalability and time. Consequently, testing capacities were limited at the initial stages in both countries. In the United Kingdom, testing commenced at a capacity of only 5,000 a day, rendering it almost impossible to make accurate estimations on national infection numbers in the early stages of the outbreak (Department of Health and Social Care, 2020). Malaysia had a testing capacity of 11,500 at the same point in time—a number more than double that of the United Kingdom (Murugesan & Harun, 2020). Interestingly, the Institute for Medical Research, MOH had already started studying and designing primers and probes specific for COVID‐19 detection on March 11 based on the genetic information of the virus China had shared with the world. This was even before WHO published a similar protocol on March 17 (DG of Health, 2020c).

The approach toward “Testing and Tracing,” however, varied greatly between both countries. Although real‐time RT‐PCR is a very specific test (test specificity 95 percent), it is reported to have a relatively high false‐negative rate, that is, a significant probability of having COVID‐19 despite being tested negative (Watson et al., 2020). Furthermore, due to the test's high sensitivity, patients who are no longer infectious, yet have not fully cleared the virus, are frequently misdiagnosed as positive, given the presence of remnants of viral RNA in their system. This, overall, creates a logistic problem where a negative test result may indeed be positive and vice versa.

Again, both countries responded differently to this challenge: in Malaysia, all individuals who tested positive were admitted to hospital for observation and/or early treatment irrespective of symptom strength. Even the asymptomatic were hospitalized for monitoring purposes (NADMA Malaysia, 2020). Those who tested negative, but showed symptoms were classified as “Persons under Surveillance” based on clinical suspicion with compulsory quarantine for 14 days, either at home or approved quarantine station. RT‐PCR was repeated on Day 13.

Unlike Malaysia, the United Kingdom implemented “at home isolation”—a strategy that may be cost‐effective but came with the trade‐off that numerous infected individuals got significantly worse before intervention was offered (National Health Service, 2020). This is particularly problematic in cases where coronavirus is known to have a worse outcome (Office for National Statistics, 2019), namely in the elderly and those with pre‐existing medical conditions. This is a pertinent point, especially when considering the two countries’ respective population demographics: England has a more elderly population with 18 percent over the age of 65 (Office for National Statistics, 2019) compared with 6.9 percent in Malaysia (Razak, 2020). Had the United Kingdom adopted the aggressive Malaysian style of test, trace, isolate and treat, it would have likely reduced mortality rates, especially among the elderly who could have received earlier intervention. Since then, the United Kingdom has focused its efforts on scaling up its testing capacity: the country is currently performing 450.48 COVID‐19 tests/1,000 people per day compared with 69.16 COVID‐19 tests/1,000 people in Malaysia (data accurate as of November 9, 2020) (Our World in Data, 2020). Furthermore, early preventive measures have recently been announced by the U.K. government to prevent, or at least limit the severity, of a second wave. These encompass tight regulations on social gatherings, for example, a maximum of 15 guests attending weddings and funerals, and restrictions on the opening hours for cafes, restaurants, and bars. Measures will be effective from September 24, likely lasting for a period of 6 months (*BBC News*, 2020). The United Kingdom has further recently introduced a three‐tier system, classifying regions within the country with an ample system according to the number of infected individual in the region in question, that is, green/Tier 1 = low risk, yellow/Tier 2 = intermediate risk, red/Tier 3 = high risk. This allows restrictions and lockdown measures to be implemented at a local and regional, rather than national level. In terms of who to test, the NHS continues offering free testing merely to symptomatic individuals and their close household contacts. Asymptomatic individuals can request COVID‐19 testing only against a fee in the private sector (gov.uk, 2020). In contrast, Malaysia has opted for “open public testing” including the asymptomatic, through “drive‐through testing” and other facilities. Comprehensive contact tracing of all confirmed cases is conducted in both counties (Our World in Data, 2020).

### Governance and Societal Compliance: The United Kingdom Versus Malaysia

Governance and coordination are crucial to any country, even more so in critical situations like the COVID‐19 pandemic. Both the United Kingdom and Malaysia were experiencing political issues at or close to the time of the outbreak. The United Kingdom, in light of discussions around Brexit, had recently had three Prime Minister changes from 2015 to 2020 (Edginton, 2020). This political instability may have resulted in insecurity amongst the population, leaving many apprehensive and distrustful toward the government. These factors may be inherently linked to societal compliance. Furthermore, the U.K. government has less power over health care since the Health and Social Care Act 2012, meaning it cannot enact quick changes to public health (The Kings Fund, 2017). Given the lack of a hierarchical system, the minister is unable to effectuate changes without prior authorization from other sectors. Although this is a sensible approach in general, it can result in a significant slow‐down of the decision making in situations where timely reactions are of the essence, like in the recent pandemic.

The Malaysian government was also facing issues as Prime Minister, Mahathir bin Mohamad, stepped down on February 24 (Ratcliffe, 2020). Following his resignation, the country found itself in a precarious situation: without a Prime Minister and functioning Cabinet, Malaysia was unable to activate the “Disease Control Act” (Act 342) for its lockdown endeavors. There was, however, a quick succession through political party realignment in the House of Representatives on March 1, 2020, leading to a new coalition governing Malaysia. Despite some opposing voices in the public, implementation was swift, involving a democratic process in alignment with the Malaysian constitution. Once the Prime Minister and Cabinet were in power, the “Disease Control Act” was successfully instated and decisions were made promptly. The National Security Council took charge of non‐health matters, while MOH was given full authority over health matters. This clear division of roles allowed for quicker implementation of public health reforms and inspired confidence in the government by the Malaysian people, and also among those initially opposing the new government. Furthermore, the central control of MOH/DG of Health over the three big functions held by the Deputy DGs and the 13 states/3 federal territories facilitated the decision making and crisis response. Unlike in the United Kingdom, many technical decisions could be made by the technocrat chief, for example, the Director‐General of Health in central committee meetings, allowing for a prompt translation into public health actions on the ground (at the district level), labs, clinics, and hospitals. A combination of these factors likely contributed to reducing the spread of COVID‐19.

A study of public views carried out by King's College London found that 9 percent of the U.K. population were resisting the quarantine (Duffy & Allington, 2020). Given the recent political uncertainty, this dark figure, however, may be much higher. In Malaysia, on the other hand, people's behavior changed even before the implementation of the MCO. People started social distancing (83.4 percent) and trusted the government (89.9 percent) (Azlan, Hamzah, Sern, Ayub, & Mohamad, 2020).

## Conclusion

The United Kingdom‘s response to Coronavirus led to 62.64 COVID‐19 deaths per million people compared to 1.79 deaths per million in Malaysia (data accurate as of November 9) (gov.uk, 2020; Our World in Data, 2020). Although multiple reasons contributed to this large disparity, it also suggests issues related to a lack of public health intervention or timeliness of its intervention. The United Kingdom, despite being more developed and economically advanced than Malaysia, fared worse in the COVID‐19 pandemic. The late response, the greater population density in the United Kingdom, the lack of testing capacity, and societal compliance at the beginning of the pandemic contributed to a substantial number of infections and fatalities. Despite having a well‐differentiated, mature health system, the U.K. failed to respond sooner to the WHO's warning, the country's deaths, and confirmed cases. Political uncertainty and inherent features of the country's health system may have contributed to this. Malaysia, by contrast, acted sooner, employing strict, effective measures. Consequently, the Malaysian population largely trusted their government and the MOH, changing their behavior to align with the new rules. Lockdown implemented at the right time, strong public health prowess of MOH at the federal level, all the way to state and local districts, seemed to have made the difference, setting Malaysia apart from the United Kingdom, despite its geographic proximity to the origin of the outbreak—China.

In recent weeks and months, however, it seems the United Kingdom has attempted to learn from its past mistakes, employing early preventive measures for the impending second wave. Nevertheless, national‐level reviews will be required during the years to come to understand how the United Kingdom's capabilities were squandered.

Undoubtedly, there are areas for improvement for both countries. It does, however, appear that the British could learn from its former colony on how to deliver better preventive public health for its people. The three main lessons learned from the COVID‐19 pandemic include the importance of

(i) acting early and allocating enough time to prepare the health‐care system,

(ii) minimizing infection numbers, and

(iii) empowerment of and trust from the public.

## Note

Logan Manikam and Shereen Allaham are funded via a National Institute for Health Research (NIHR) Advanced Fellowship (Ref: NIHR300020) to undertake the feasibility randomised controlled trial of the NEON study in East London.

**Corresponding author**: Shereen Allaham, s.laham@ucl.ac.uk
